# Effects of a Serious Game for Adolescent Mental Health on Cognitive Vulnerability: Pilot Usability Study

**DOI:** 10.2196/47513

**Published:** 2024-05-09

**Authors:** Eva De Jaegere, Kees van Heeringen, Peter Emmery, Gijs Mommerency, Gwendolyn Portzky

**Affiliations:** 1 Department of Head and Skin, Faculty of Medicine and Health Sciences, Ghent University, Ghent, Belgium; 2 University Psychiatric Centre KU Leuven, KU Leuven, Leuven, Belgium; 3 Department of Child and Adolescent Psychiatry, Ghent University Hospital, Ghent, Belgium

**Keywords:** e-health, cognitive psychology, cognitive distortion, cognitive vulnerability, digital health, serious games, adolescent mental health, prototype, adolescent, prevention, eHealth

## Abstract

**Background:**

Adolescent mental health is of utmost importance. E-mental health interventions, and serious games in particular, are appealing to adolescents and can have beneficial effects on their mental health. A serious game aimed at improving cognitive vulnerability (ie, beliefs or attitudes), which can predispose an individual to mental health problems, can contribute to the prevention of these problems in adolescents.

**Objective:**

This study aimed to assess the feasibility of the prototype of a serious game called “Silver.”

**Methods:**

The prototype of the serious game was developed using a user-centered participatory design. The prototype of Silver focused on 1 aspect of a serious game for improving cognitive vulnerability in adolescents, that is, the recognition and identification of cognitive distortions. Through the game, players were required to identify and classify the character’s thoughts as helpful or unhelpful. Upon successful advancement to the next level, the task becomes more challenging, as players must also identify specific types of cognitive distortions. A pre- and posttest uncontrolled design was used to evaluate the game, with a 1-week intervention phase in which participants were asked to play the game. Participants aged 12-16 years were recruited in schools. The outcomes of interest were the recognition of cognitive distortions and presence of participants’ cognitive distortions. The game was also evaluated on its effects, content, and usefulness.

**Results:**

A total of 630 adolescents played Silver and completed the assessments. Adolescents were significantly better at recognizing cognitive distortions at the pretest (mean 13.09, SD 4.08) compared to the posttest (mean 13.82, SD 5.09; *t*_629_=−4.00, *P*<.001). Furthermore, their cognitive distortions decreased significantly at the posttest (mean 38.73, SD 12.79) compared to the pretest (mean 41.43, SD 10.90; *t*_629_=7.98, *P*<.001). Participants also indicated that the game helped them recognize cognitive distortions. Many participants considered the game appealing (294/610, 48.2%) but boring (317/610, 52%) and preferred a more comprehensive game (299/610, 49%).

**Conclusions:**

Findings from this study suggest that a serious game may be an effective tool for improving cognitive vulnerability in adolescents. The development of such a serious game, based on the prototype, is recommended. It may be an important and innovative tool for the universal prevention of mental health problems in adolescents. Future research on the effects of the game is warranted.

## Introduction

Mental illness is one of the biggest health burdens worldwide, and adolescent mental health is a particular global concern [1]. According to the United Nations’ recently published report on children’s mental health, approximately 1 in 7 adolescents aged between 10 and 19 years experience a diagnosed mental disorder globally [2]. Approximately 40% of these disorders are attributable to anxiety and depression [1]. The psychological distress and economic costs due to adolescent mental health problems are enormous. In 2021, the invisible economic cost due to mental health problems in adolescents was estimated at US $387 billion per year globally [2]. Moreover, these mental health problems are important risk factors for self-harm and suicide among adolescents [3,4].

Early access to treatment can improve outcomes, but limited treatment resources result in long waiting lists and undertreatment [5]. Society, and health systems in particular, need to cope with the increasingly high demands to reduce costs and prevent self-harm and suicide among adolescents. E-mental health interventions are already being used across health care and may be more accessible, engaging, and acceptable options [6-8]. These can take various forms, such as text-based programs, multimedia and interactive programs, virtual reality–based programs, and serious games [9-12]. Evidence of their beneficial effects is increasingly provided [6,13-15]. Regarding serious games, a recent review concluded that the limited evidence indicates a beneficial effect on reducing mental health problems [11,16-19]. Thus, there is a massive potential for serious games to be a new, emerging treatment modality that is more acceptable and engaging, as it uses game mechanics, especially for adolescents who are major users of computerized games in the present-day digital world. However, more research is needed [11,18,20].

This study therefore aimed at providing further evidence of the beneficial effects of serious games by piloting a prototype (“Silver”) developed to counter cognitive characteristics, which are known to increase the vulnerability to common mental health problems such as depression and anxiety [21]. More specifically, the prototype targets adolescents (aged 12-16 years), aiming to gain insight into cognitive distortions (ie, negative, biased thoughts that influence people’s interpretation of themselves or the world [22]) and their effect on feelings and behaviors. Cognitive distortions, including all-or-nothing thinking, overgeneralization, or mind reading, can contribute to the development of mental health issues such as depression or anxiety during adolescence [23-25]. Identifying and modifying these distortions and replacing them with more balanced, helpful thoughts—that is, cognitive restructuring—is a common technique used in cognitive behavioral therapy (CBT) [22], which has been demonstrated to prevent mental health issues during this vulnerable development phase [26,27] and improve mental well-being [28]. Given the relationship between cognitive distortions and adolescent mental health, as well as the potential of CBT-based techniques to address these distortions, this study aimed to investigate the specific impact of the prototype on adolescents’ cognitive processing. Therefore, the primary hypothesis was that adolescents would improve at recognizing and categorizing helpful and unhelpful thoughts (ie, cognitive distortions) after playing Silver. Furthermore, playing the game was expected to lead to a decline in cognitive distortions. Finally, the usability of Silver was assessed, focusing on the appeal of the game.

## Methods

### Participants and Recruitment

Participants were aged between 12 and 16 years and had a smartphone or tablet. Participants were excluded if they were not proficient in Dutch. Recruitment took place from August 2017 to October 2017 via schools.

### Ethical Considerations

School directors of 8 secondary schools with different curricula across Flanders (ie, the Dutch-speaking region in Belgium) consented to participate in the study. Parents or guardians were informed about the study and given the opportunity to decline their child’s participation (opt out). Web-based assent was obtained from the adolescents before the start of the study. Participants did not receive any form of compensation for their involvement in this study. The data were deidentified prior to analysis to safeguard participants’ privacy. The study was approved by the Commission for Medical Ethics of the University Hospital Ghent (Belgian registration B670201731975).

### Design and Procedure

The prototype was evaluated using a pre- and posttest uncontrolled design with a 1-week intervention phase. Before participants received access to the prototype, they were asked to fill in a web-based questionnaire (pretest). Immediately after completing the questionnaire, they received access to Silver, which they were asked to play daily for 1 week. After 1 week, they were asked to fill in the second web-based questionnaire (posttest).

### Intervention

The prototype of the serious game Silver aims to reduce cognitive distortions in adolescents. The prototype is based on a cognitive behavioral framework and focuses on 1 element of mental health improvement, that is, gaining insight into cognitive distortions and their effects on feelings and behaviors. The prototype was designed and developed in a cocreative manner, in which the target users themselves (ie, adolescents aged 12-16 years) were involved, as well as a clinical child psychologist, a child and adolescent psychiatrist, and professional game designers. The design, therefore, was user centered and participatory. The cocreation process was managed by the company that developed the game’s prototype.

**Figure 1.**
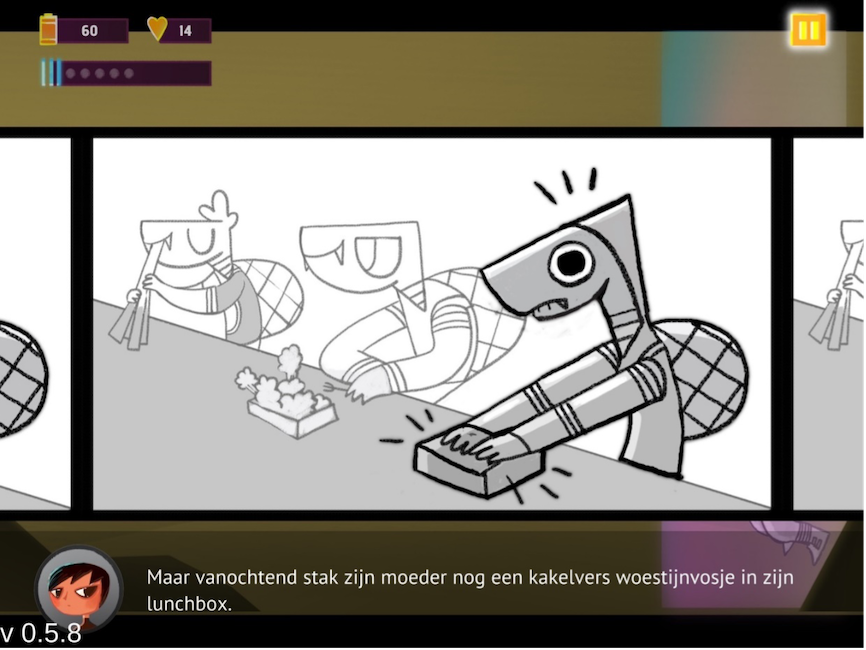
Screenshot of Silver, showing a flashback to the incident before the glitch. “Maar vanochtend stak zijn moeder nog een kakelvers woestijnvosje in zijn lunchbox.” means “But this morning, his mother stuck another brand-new desert fox in his lunch box.”

**Figure 2.**
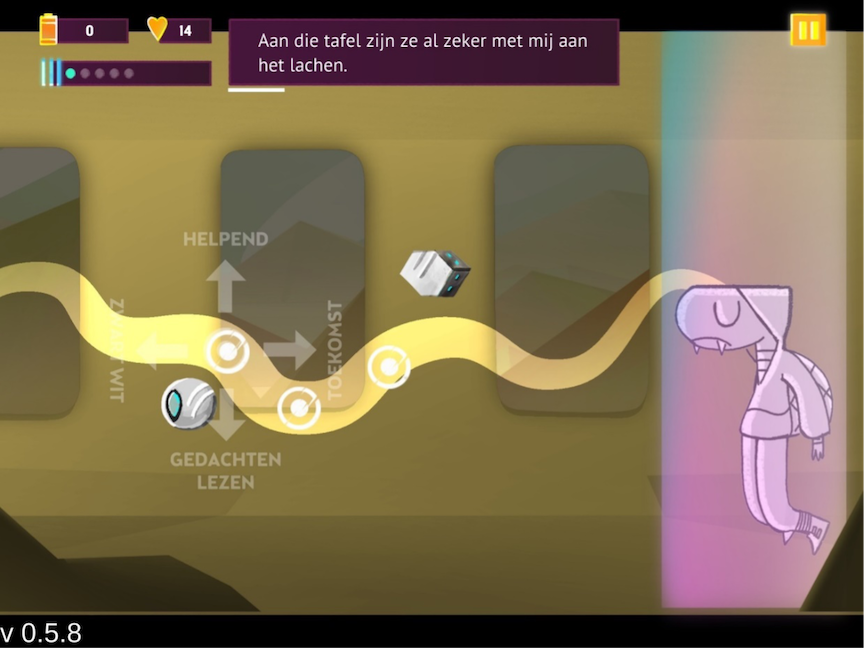
Screenshot of Silver, showing the process of identifying types of cognitive distortions. “Aan tafel zijn ze al zeker met mij aan het lachen.” means “At the table, they are already definitely laughing at me.“; “Helpend” means “Helpful”; “Toekomst” means ”Future”; “Gedachten lezen” means “Read minds”; and “Zwart-wit” means “Black and white.”

The game is set up in 3 different worlds inhabited by anthropomorphic animals. Each world has different chapters that can be played. The more a player progresses in the game, the more difficult it becomes. The game always starts with an animal that is stuck in his or her mind and therefore is in a “glitch.” The incident preceding the “glitch” is explained through a flashback where the player is shown how this came about (see Figure 1). The events represent difficult situations that are very relatable for adolescents (eg, not getting likes on a social media post). Afterward, the player is shown the character’s thoughts. Each time after reading a thought, the player must decide whether it is a helpful or unhelpful thought (ie, cognitive distortion). If the thoughts are correctly recognized, the unhelpful thoughts are fired upon by little robots and the helpful thoughts return to the character’s head. When enough helpful thoughts have been collected, the character is released from his or her “glitch” and the chapter is completed. At higher levels, the player is also asked to indicate which type of unhelpful thought it is. At first, 2 types of cognitive distortions are introduced, that is, future thinking and all-or-nothing thinking. Afterward, 1 more type of cognitive distortion is added, that is, mind reading (see Figure 2). Thus, the further you progress through the game, the more difficult it becomes as the thoughts can be categorized into more types of cognitive distortions. In this way, the player learns to gain insight into the different types of cognitive distortions.

### Measures

All outcome measures were collected via self-report questionnaires, which were administered on the web.

#### Demographics

Sociodemographic information (ie, gender, age, and education) was assessed at the pretest. Data on sex (male, female, or other), age (in years), and education (first or second grade and type of curriculum, ie, general secondary education, technical secondary education, secondary education in the arts, and vocational education) were obtained. In addition, participants were asked if they had ever been in therapy for psychological problems.

#### Media and Game Use

At baseline, data were collected regarding participants’ use of media by asking participants what type of media they used and how often they used it on a 5-point scale (1=“never”; 5=“daily”). Items regarding game use assessed whether they ever played computer or video games and whether they still played them. Furthermore, participants were asked on which devices they played the games and how often they did this on a 5-point scale (1=“never”; 5=“daily”). They also gave an estimate about their knowledge about games, ranging from 1= “no knowledge” to 4=“expert.”

#### Primary Outcome Measure: Recognition of Negative and Positive Automatic Thoughts

The primary outcome measure focused on recognizing helpful and unhelpful thoughts. A questionnaire was developed, in which 20 items of the Dysfunctional Attitude Scale, Form A, Dutch translation [29,30] were used. Participants were asked to classify each item as helpful, unhelpful, or “I do not know.” Items were scored as true or false. Scores ranged from 0 to 20, with higher scores indicating a better identification of negative and positive automatic thoughts. The internal consistency of the scale in this study was α=.82.

#### Secondary Outcome Measure: Presence of Cognitive Distortions

The Children’s Negative Cognitive Error Questionnaire–Revised [31] is a 16-item self-report questionnaire that assesses cognitive distortions in those aged 9-17 years. The questionnaire consists of 5 subscales that measure 5 categories of cognitive distortions: “underestimation of the ability to cope,” “personalizing without mind reading,” “mind reading,” “selective abstraction,” and “overgeneralizing.” In each item, a situation is described, followed by a possible thought about the situation. Participants are asked to rate on a 5-point Likert scale how much the thought corresponds to what they would think in that situation, ranging from “almost exactly like I would think” (5 points) to “not at all like I would think” (1 point). Total scores range from 16 to 80, with a higher score reflecting more distorted cognitive errors. The total scale has a good level of internal consistency and good test-retest reliability [31,32].

#### Game Evaluation

At the posttest, participants were asked to rate various statements regarding the effects, content, and usefulness of the game. Items were rated on a 5-point Likert scale (1=“completely disagree”; 5=“completely agree”). An example of an item is “By playing the game I will be able to recognize my own cognitive distortions.” Participants were also asked how they would rate the game overall on a scale from 0 to 10.

### Statistical Analysis

Power and sample size could not be based on previous studies due to a lack of comparable studies. An effect size of 0.3 was assumed. To detect such an effect size with α=.05 and β=.80, a total sample of 500 participants was calculated. However, since a possible high dropout of 70% to 75% was expected [33], the total required sample size was estimated at 1753.

Differences between the participants of the study and those who dropped out during the study, as well as differences between those who played the game (ie, gamers) and those who did not play it (ie, nongamers), were examined with *χ*^2^ tests (for categorical variables) and 2-tailed independent-sample *t* tests (for continuous variables). Mean changes between the pre- and posttests were carried out using 2-tailed paired-samples *t* tests. The corresponding effect sizes were assessed using Cohen *d*. A significance level of .05 was used for all outcome analyses. All data were analyzed using SPSS software (version 27; IBM Corp).

## Results

### Sociodemographic Characteristics and Baseline Outcome Measure

A total of 1654 adolescents signed up to take part in the study. Among these, 1140 took part in the pre- and posttests. Table 1 presents the differences between the participants who completed both the pre- and posttests and those who only completed the pretest.

**Table 1.** Sociodemographic characteristics and baseline outcome measures of participants who completed both the pre- and posttests and participants who only completed the pretest.

Characteristics		Pre- and posttests (n=1140)		Pretest only (n=514)		*P* value
**Sex, n (%)**						
	Female	700 (61.4)		303 (58.9)		.34
	Male	429 (37.6)		206 (40.1)		.34
	Other	11 (1)		5 (1)		.59
Age (y), mean (SD)		13.40 (1.32)		13.86 (1.36)		.001
**Education curriculum, n (%)**						
	First-grade GSE^a^	306 (26.8)		84 (16.4)		<.001
	First-grade VE^b^	55 (4.8)		79 (15.4)		<.001
	Second-grade GSE	513 (45)		158 (30.8)		<.001
	Second-grade TSE^c^	173 (15.2)		56 (10.9)		.02
	Second-grade SEA^d^	20 (1.8)		51 (9.9)		<.001
	Second-grade VE	73 (6.4)		85 (16.6)		<.001
**Treatment for psychological problems, n (%)**						
	Never been to therapy	984 (86.3)		419 (81.7)		.02
	More than a year ago	68 (6)		32 (6.2)		.83
	Less than a year ago	50 (4.4)		31 (6)		.15
	In therapy	38 (3.3)		31 (6)		.01
**Baseline outcome measures, mean (SD)**						
	Recognizing automatic thoughts	12.64 (4.37)		11.51 (4.82)		<.001
	CNCEQ-R^e^	40.81 (10.97)		40.66 (12.13)		.82

a GSE: general secondary education.

b VE: vocational education.

c TSE: technical secondary education.

d SEA: secondary education in the arts.

e CNCEQ-R: Children’s Negative Cognitive Error Questionnaire–Revised.

Of the 1140 adolescents who completed the pre- and posttests, 510 (44.7%) reported that they did not engage with the game (hereafter referred to as nongamers). The primary reason for nonengagement was technical problems (233/510, 45.7%) such as inability to download the game. Other reasons included a lack of time or forgetfulness (198/510, 38.8%), a disinterest in the game (51/510, 10%), and other unspecified reasons (59/510, 5.5%). In contrast, 630 (55.3%) of the 1140 adolescents indicated that they played the game (hereafter referred to as gamers). There were no significant differences in all baseline sociodemographic characteristics between gamers and nongamers except for type of education curriculum (*P*=.02; see Table 2). Additionally, adolescents who played the game scored significantly higher on recognizing automatic thoughts (mean 13.09, SD 4.08 vs mean 12.08, SD 4.65; *t*_1020_=–3.86, *P*<.001) and significantly higher on the Children’s Negative Cognitive Error Questionnaire–Revised (mean 41.43, SD 10.90 vs mean 40.05, SD 11.01; *t*_1138_=–2.10, *P*=.04) at baseline (see Table 2).

**Table 2.** Sociodemographic characteristics and baseline outcome measures of gamers and nongamers in schools.

Characteristics		Gamers (n=630)		Nongamers (n=510)		*P* value
**Sex, n (%)**						
	Female	371 (58.9)		329 (64.5)		.053
	Male	251 (39.8)		178 (34.9)		.09
	Other	8 (1.3)		3 (0.6)		.24
Age (y), mean (SD)		13.43 (1.30)		13.36 (1.33)		.40
**Education curriculum, n (%)**						
	First-grade GSE^a^	186 (29.5)		120 (23.5)		.02
	First-grade VE^b^	25 (4)		30 (5.9)		.13
	Second-grade GSE	264 (41.9)		249 (48.8)		.02
	Second-grade TSE^c^	99 (15.7)		74 (14.5)		.57
	Second-grade SEA^d^	14 (2.2)		6 (1.2)		.18
	Second-grade VE	42 (6.7)		31 (6.1)		.69
**Treatment for psychological problems, n (%)**						
	Never been to therapy	542 (86)		442 (86.7)		.76
	More than a year ago	39 (6.2)		29 (5.7)		.72
	Less than a year ago	31 (4.9)		19 (3.7)		.33
	In therapy	18 (2.9)		20 (3.9)		.32
**Baseline outcome measures, mean (SD)**						
	Recognizing automatic thoughts	13.09 (4.08)		12.08 (4.65)		<.001
	CNCEQ-R^e^	41.43 (10.90)		40.05 (11.01)		.04

a GSE: general secondary education.

b VE: vocational education.

c TSE: technical secondary education.

d SEA: secondary education in the arts.

e CNCEQ-R: Children’s Negative Cognitive Error Questionnaire–Revised.

### Media and Game Use

Table 3 shows media and game use of the gamers versus the nongamers. The nongamers were significantly less likely to play games currently (270/510, 52.9% vs 405/630, 64.3%; *χ*^2^_1_=15.02, *P*<.001) and were significantly more likely to have “no knowledge” about games than the gamers (69/510, 13.5% vs 54/630, 8.6%; *χ*^2^_1_=7.20, *P*=.007).

**Table 3.** Media and game use of gamers and nongamers in schools.

Media and game use		Gamers (n=630)	Nongamers (n=510)	*P* value
**Media use, n (%)**				
	Smartphone use	615 (97.6)	496 (97.3)	.70
	Tablet use	466 (74)	360 (70.6)	.20
	Desktop use	226 (35.9)	179 (35.1)	.79
	Laptop use	501 (79.5)	409 (80.2)	.78
	Game console use	367 (58.3)	279 (54.7)	.23
**Game playing, n (%)**				
	Ever	601 (95.4)	476 (93.3)	.13
	Currently	405 (64.3)	270 (52.9)	<.001
**Game knowledge, n (%)**				
	No knowledge	54 (8.6)	69 (13.5)	.007
	Beginner	260 (41.3)	211 (41.4)	.97
	Advanced	255 (40.5)	198 (38.8)	.57
	Expert	61 (9.7)	32 (6.3)	.04

### Outcome Measures

Table 4 shows the mean changes in the outcome measures from pre- to posttest and its effect sizes for the group that played the game. The gamers significantly improved in recognizing automatic thoughts (*P*<.001) and had significantly fewer distorted cognitive errors (*P*<.001). On both measures, the difference represented a small effect size (0.16 and 0.23, respectively).

**Table 4.** Pre- and posttest scores on outcome measures and 2-tailed paired *t* test results.

Outcome measures	Pretest score, mean (SD)	Posttest score, mean (SD)	*t* test (*df*)	*d* (95% CI)	*P* value
Recognizing automatic thoughts	13.09 (4.08)	13.82 (5.09)	−4.00 (629)	0.16 (−1.09 to −0.37)	<.001
CNCEQ-R^a^	41.43 (10.90)	38.73 (12.79)	7.98 (629)	0.23 (3.36 to 7.98)	<.001

a CNCEQ-R: Children’s Negative Cognitive Error Questionnaire–Revised.

### Game Evaluation

A total of 610 gamers gave a score on the various evaluation items (see Table 5). Besides the high number of neutral responses, they generally moderately or highly agreed with the items. The median overall satisfaction rating of 599 gamers, which was scored on a scale of 1 to 10, was 6 and the mean was 5.51 (SD 2.30).

**Table 5.** Game evaluation ratings (n=610).

Statements	Disagree, n (%)	Neutral, n (%)	Agree, n (%)	Score, mean (SD)
By playing the game I learned about different ways of thinking.	134 (22)	278 (45.6)	198 (32.5)	2.10 (0.73)
By playing the game I will recognize my unhelpful thoughts.	123 (20.2)	244 (40)	243 (39.8)	2.20 (0.75)
The game helps me to reflect more upon my thoughts.	164 (26.9)	272 (44.6)	174 (28.5)	2.02 (0.75)
I can empathize with the stories in the game.	202 (33.1)	204 (33.4)	204 (33.4)	2.00 (0.82)
I think the game is beautifully made.	96 (15.7)	220 (36.1)	294 (48.2)	2.32 (0.73)
I find the game difficult.	283 (46.4)	198 (32.5)	129 (21.1)	1.75 (0.78)
I find the game boring.	122 (20)	171 (28)	317 (52)	2.32 (0.79)
I would like it if the game was not only about thoughts but also about feelings and relaxation.	102 (16.7)	209 (34.3)	299 (49)	2.32 (0.74)

## Discussion

### Principal Findings

The aim of this pilot study was to test the efficacy and usability of the prototype of Silver, a serious game aimed at reducing cognitive distortions in adolescents and thus decreasing cognitive vulnerability to mental health problems. The trial supports our hypothesis that automatic thoughts are better recognized after playing Silver. This enhancement in cognitive awareness was also reflected in the evaluation of the game, wherein gamers indicate that playing the game helps in recognizing unhelpful thoughts. Furthermore, after having played the game, adolescents showed fewer cognitive distortions than before playing. These findings are in line with prior studies, underscoring the positive impact of serious games on cognitive beliefs and modification [19,34-37]. This study contributes to the growing evidence on digital interventions that incorporate core components of CBT, such as cognitive restructuring, and their beneficial effects on well-being and mental health issues [38]. Silver’s emphasis on identifying and mitigating cognitive distortions aligns closely with the principles of rational emotive behavior therapy (REBT), a type of CBT [39]. This therapeutic approach focuses on the identification, challenge, and substitution of irrational beliefs with rational counterparts, alongside learning to manage emotions and behavior in a more helpful way. Literature suggests that serious games focusing on REBT techniques seem to have a strong positive effect in mitigating symptoms of depression and anxiety [19]. Considering the significant outcomes associated with the current version of Silver, which primarily targets cognitive distortions, an expanded version of the game that includes elements intended to address emotional and behavioral aspects could potentially have a greater positive impact on mental health. The inclusion of these additional components could further enhance the game’s therapeutic effectiveness, aligning with REBT’s approach to mental health improvement [19].

Regarding the evaluation of Silver, the majority (294/610, 48.2%) of gamers indicated that Silver is appealing. Research has shown that nonappealing interfaces may be off-putting and may cause adolescents to disengage from the game. The cocreation of Silver with the target audience probably has ensured that it has attractive aesthetics [40]. In contrast, the majority (317/610, 52%) also perceived Silver as boring. Adolescents may experience serious games in such a manner since they often have very didactic content that does not match commercial, off-the-shelf games, and as a result, the adolescents may cease playing these games [40]. This dichotomy can be attributed to the prototype’s focus on a single aspect of cognitive vulnerability (ie, cognitive distortions), which, although important, may lack the variety necessary to sustain players’ interest over time. Therefore, this may be a critical area for further development in diversifying the game’s content and mechanics. The game could be broadened to include a range of elements. This could mitigate the issue of monotony, thereby improving overall engagement and effectiveness [19]. Furthermore, the gamers indicated that they would like a more comprehensive game that also deals with feelings and relaxation. Such a game may be more eventful, more fascinating, and less boring.

Preceding a discussion of potential implications of the study findings for the development of serious games, methodological issues need to be addressed. First, as this was a pre- and posttest study, a control group was lacking. Therefore, we were unable to compare the effects on the gamers with those in a random control group that did not play the game. A convenience sample was used, which can lead to a selection bias and consequently underrepresent or overrepresent particular groups. Efforts were made to counter this as much as possible by recruiting a large number of adolescents from 8 different schools with various curricula. However, it is also unclear why some adolescents agreed to take part in the study but others did not. As the sample was not chosen at random, the inherent bias in convenience sampling means that any generalizations of findings must be made with caution. Second, a large group did not adhere to the study protocol. The main reason for dropout were technical difficulties. These were largely due to the prototypic nature of our app; as it was not readily available on app stores, it required adolescents to undertake multiple steps before receiving access to the game. Moreover, participants’ feedback showed time constraints and forgetfulness as additional factors for not engaging with the game. To mitigate these issues in future studies, it is imperative to streamline the app’s accessibility, potentially by securing its availability on common digital distribution platforms. Furthermore, incorporating human support may serve to enhance participant engagement and possibly the overall effectiveness of the intervention [41]. Addressing these aspects is critical for improving study adherence and ensuring the robust evaluation of the app’s therapeutic potential. Furthermore, participants who dropped out were older and had a lower education level. Additionally, they may have experienced more mental health problems, as baseline measurements showed that they were less skilled at recognizing automatic thoughts and were currently more likely to be in treatment for psychological problems. The nongamer group encompassed more adolescents with little interest in games, as they currently played no games or had less knowledge about them. In addition, they were less skilled at recognizing automatic thoughts but had fewer cognitive distortions themselves. However, high attrition rates are not uncommon when studying e-mental health interventions. A systematic review and meta-analysis of computer-based psychological treatments showed an overall dropout rate of 57%, which further increased to 74% in unsupported digital programs [6,42]. In this study, the adolescents were also not offered any support, and this may have had a major effect on adherence. Adding human support could decrease the attrition rate and may even increase the effectiveness of the serious game [43,44]. Moreover, the effectiveness of serious games is often assessed in pragmatic study trials. The real-life settings in which these studies are carried out can also have an impact on the attrition rate. Nevertheless, this type of study can improve the generalizability of the results, as the environments in which they will be implemented are similar to the ones in the trials [45]. Third, no standardized questionnaire for the recognition of automatic thoughts was used. The questionnaire was based on an existing standardized questionnaire [29,30] but was adapted for this study. Fourth, participants often responded with “neutral” in the game evaluation. Although they may have used the neutral midpoint response because they did not comprehend the items or were undecided, offering these neutral responses may decrease the quality and reliability of a questionnaire, particularly in adolescents, since they are more sensitive to pleasing by selecting a neutral answer. Future studies should consider omitting the neutral midpoint [46,47]. Fifth, the study’s emphasis on quantitative measures may have introduced acquiescence bias. Adding qualitative methodologies may provide a more nuanced perspective of the participants’ experiences with the prototype. Future studies should consider using a mixed methods approach to enhance understanding of the intervention’s impact [48].

Lastly, the eligibility criterion requiring participants to possess a smartphone or tablet may have introduced a selection bias, potentially excluding adolescents without access to such technology or those reluctant to use it. This could inadvertently reinforce the digital divide, that is, inequalities in accessing and using information and communication technologies [49], and limit the generalizability of the findings. Future research should address this limitation by using more inclusive recruitment strategies to minimize technological barriers and ensure broader participation.

It is difficult to assess the effect of these methodological issues on the validity of the current findings, more so as the current results are difficult to compare to those from similar previous studies. The few available studies of serious games aimed at cognitive training had targeted adults or children with particular mental health problems such as anxiety, alcohol use disorder, or attention-deficit/hyperactivity disorder [11,20]. To the best of the authors’ knowledge, only 2 serious games were studied regarding effects on emotional resilience or mental health promotion in a community sample of adolescents. The results of these studies are comparable to our findings [20,35,36].

### Conclusions

In conclusion, and keeping the limitations mentioned above in mind, this pilot study demonstrates the promising effects of the Silver prototype. Notably, participants exhibited not only an enhanced ability to recognize cognitive distortions but also a significant decrease in their own experiences of such distortions after engaging with the prototype. This observation suggests a potential positive influence on cognitive characteristics, which are commonly associated with mental health issues. It is therefore recommended that a serious game aimed at decreasing cognitive vulnerability and therefore improving mental health in a general population of adolescents should be developed further and that its efficacy should be studied in future research. This study provides a few cues for further research. The dropout of adolescents who may have the greatest need for cognitive restructuring is a matter of concern, and reasons and remedies for this worrisome issue should be targeted in future research. Randomized controlled trials should be used to further explore the effects of serious games on adolescents in the general population, preferably using an active control group that engages with a different type of digital intervention [50]. Follow-up periods should be sufficiently long to study potential preventative effects among adolescents (Reynard et al [20]).

## Abbreviations

CBT: cognitive behavioral therapy
REBT: rational emotive behavior therapy
